# P-809. Clinical Impact of Novel, Rapid, Phenotypic Antimicrobial Susceptibility Testing System from Positive Blood Cultures

**DOI:** 10.1093/ofid/ofaf695.1017

**Published:** 2026-01-11

**Authors:** George W Jowsey, Matthew Spence, Rebecca Yee, Jose Lucar

**Affiliations:** George Washington University School of Medicine and Health Sciences, Washington, DC; George Washington University Hospital, Washington, District of Columbia; George Washington University School of Medicine and Health Sciences, Washington, DC; The George Washington University, Washington, DC

## Abstract

**Background:**

Antimicrobial susceptibility testing (AST) directly from positive blood culture (PBC) bottles allows for faster optimization of antimicrobial therapy, which may lower morbidity and mortality, shorten hospital length of stay, and reduce drug side effects. The Q-linea ASTar is a fully automated, phenotypic AST system from PBCs. We evaluated the accuracy and potential clinical impact of the ASTar in a cohort of patients with Gram-negative (GN) bacteremia.

**Methods:**

Seventy-five GN PBC bottles from unique patients were evaluated. AST results from the ASTar (BC G- IUO panel) were compared to AST results from standard of care overnight subculture from PBC bottles (using the Microscan Walkaway plus System). Retrospective chart review was performed to collect pertinent medical history, antimicrobial history, turnaround time of AST results, and determine the hypothetical impact on clinical outcomes.

**Results:**

The patients were located in the inpatient wards (46%), ED (28%), and ICU (26%) at the time of blood culture collection. A majority were diagnosed with GN bloodstream infections with known primary source (58%), and 42% had sepsis without septic shock. The ASTar yielded essential and categorical agreement of 95.5% and 91.8%, respectively. Implementation of the ASTar system decreased the time-to-phenotypic AST after initial positive Gram-stain result by 38.8 hours compared to the standard of care workflow (p< 0.00001). Using the results generated by ASTar, common antimicrobials administered would have been ceftriaxone (32%), cefepime (21%), fluoroquinolones (15%), and meropenem (13%). Optimized changes to targeted therapy would be seen in 78% of the patients (de-escalation:53%, route of administration adjustment:19%, and escalation:15%). Clinical outcomes that could have been impacted included exposure to fewer antibiotics (51%), discharge with oral antibiotics (24%), fewer side effects (19%), and decreased length of stay (19%).

**Conclusion:**

The Q-linea ASTar system generated reliable AST results and can accelerate phenotypic AST results by >24 hours. Technologies accelerating AST results combined with hospital antimicrobial stewardship efforts continue to show promise in improving antimicrobial use and patient outcomes.

**Disclosures:**

Rebecca Yee, PhD, BioMerieux: Grant/Research Support|Q-linea: Grant/Research Support|Q-linea: Honoraria

